# Laboratory Effects of COVID-19 Infection in Pregnant Women and Their Newborns: A Systematic Review and Meta-Analysis

**DOI:** 10.3389/fgwh.2021.647072

**Published:** 2021-04-13

**Authors:** Clark Zhang, Haitao Chu, Y. Veronica Pei, Jason Zhang

**Affiliations:** ^1^Division of Biostatistics, University of Minnesota, Minneapolis, MN, United States; ^2^Department of Emergency Medicine, University of Maryland, Baltimore, MD, United States

**Keywords:** COVID-19, preterm birth, neonatal sepsis, meta-analysis, blood assay, vertical transmission, passive immunity

## Abstract

Amidst the COVID-19 pandemic, there is a need for further research on its manifestation in pregnant women, since they are particularly prone to respiratory pathogens, like severe acute respiratory syndrome coronavirus 2 (SARS-CoV-2), due to physiological changes during pregnancy. Its effects on infants born to mothers with COVID-19 are also not well-studied, and more evidence is needed on vertical transmission of the disease from mother to infant and on the transmission of IgG/IgM antibodies between mother and infant. We aim to systematically review and evaluate the effects of COVID-19 among SARS-CoV-2-positive pregnant women in late pregnancy and neonates with SARS-CoV-2-positive pregnant mothers using blood assays to find indicators of maternal and neonatal complications. We searched for original published articles in Google Scholar, Medline (PubMed), and Embase databases to identify articles in the English language from December 2019 to July 20, 2020. Duplicate entries were searched by their titles, authors, date of publication, and Digital Object Identifier. The selected studies were included based on patient pregnancy on admission, pregnant mothers with laboratory-confirmed COVID-19 virus, maternal/neonatal complications, and blood test results. We excluded duplicate studies, articles where full text was not available, other languages than English, opinions, and perspectives. The meta-analysis using the Generalized Linear Mixed model was conducted using the “meta” and “metaprop” packages in R code. Of the 1,642 studies assessed for eligibility, 29 studies (375 mothers and neonates) were included. Preterm birth rate was 34.2%, and cesarean section rate was 82.7%. Maternal laboratory findings found elevated neutrophils (71.4%; 95% CI: 38.5–90.9), elevated CRP (67.7%; 95%: 50.6–81.1), and low hemoglobin (57.3%; 95% CI: 26.0–87.8). We found platelet count, lactate dehydrogenase, and procalcitonin to be less strongly correlated with preterm birth than between high neutrophil counts (*P* = 0.0007), low hemoglobin (*P* = 0.0188), and risk of preterm birth. There is little evidence for vertical transmission. Elevated procalcitonin levels (23.2%; 95% CI: 8.4–49.8) are observed in infants born to mothers with COVID-19, which could indicate risk for neonatal sepsis. These infants may gain passive immunity to COVID-19 through antibody transfer via placenta. These results can guide current obstetrical care during the current SARS-CoV-2 pandemic.

## Introduction

A global pandemic due to the outbreak of a novel coronavirus was first reported in Wuhan, China in December 2019. This novel coronavirus, the severe acute respiratory syndrome coronavirus 2 (SARS-CoV-2), causes the disease COVID-19. While the name of SARS-CoV-2 suggests that COVID-19 is primarily a respiratory illness presenting with symptoms including fever, cough, and shortness of breath, which may progress to respiratory failure, COVID-19 can present with a wide spectrum of symptoms including sore throat, headache, loss of taste or smell, nausea, vomiting, and diarrhea (1).

Pregnant women are particularly prone to respiratory pathogens, like SARS-CoV-2, due to physiological changes during pregnancy; increased oxygen intake and diaphragm elevation make pregnant women susceptible to hypoxia (2, 3). There is accumulating evidence on pregnant women with COVID-19. However, early data do not indicate that pregnant women are at increased risk of morbidity, but do indicate increased risk for ICU admission and ventilation (4). Furthermore, because of increased concentration of ACE2 receptors in the placenta, there is concern about the possibility of vertical transmission from mother to infant (5). In fact, case studies have shown that SARS-CoV-2 can infect the placenta (6, 7); this study investigated whether or not this risk of vertical transmission is significant.

Reported laboratory abnormalities seen in pregnant patients with COVID-19 include lower white blood cell counts (lymphopenia and thrombocytopenia) and increased C-reactive (CRP) protein levels, elevated lactate dehydrogenase, and prolonged prothrombin time (8, 9). To investigate these abnormalities, we systematically reviewed the blood assays among SARS-CoV-2-positive pregnant women in late pregnancy and among neonates with SARS-CoV-2-positive pregnant mother to find indicators of maternal and neonatal complications. We compared these laboratory values to those of non-COVID-19-infected pregnant women and to those of non-pregnant women to provide more accurate diagnosis. By identifying laboratory indicators and trends in preterm birth, neonatal sepsis, and other complications, clinicians are better prepared for treating those complications before they manifest.

## Methods

This systematic review and meta-analysis were performed according to the Preferred Reporting Items for Systematic Reviews and Meta-Analyses (PRISMA) guidelines for reviews of analytical observational studies.

### Search Strategy and Eligibility Criteria

**Figure 1:**
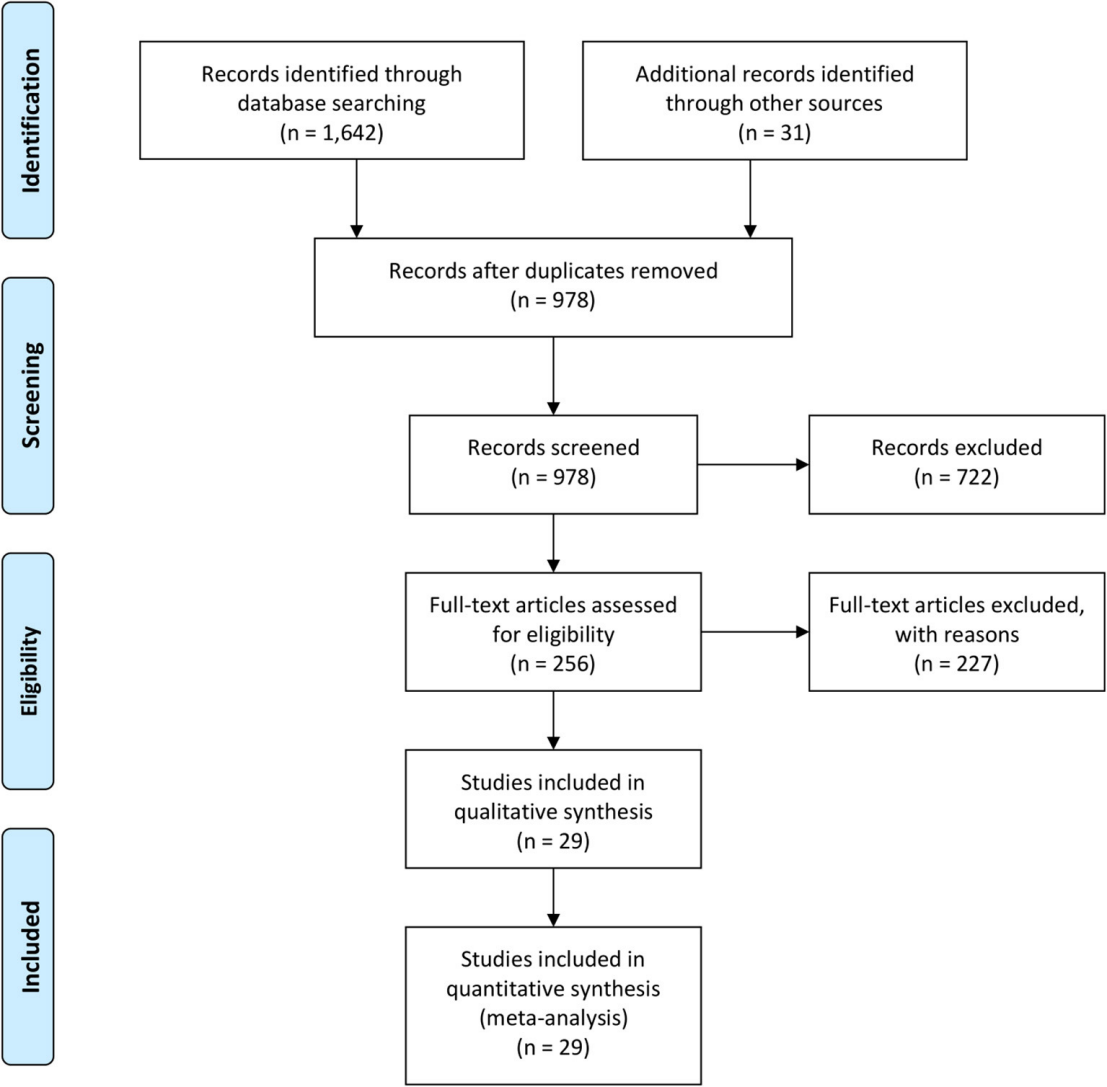
PRISMA flow diagram.

We searched for original published articles in Google Scholar, Medline (PubMed), and Embase databases to identify articles reporting maternal and neonatal complications in pregnant women with COVID-19 in accordance with the PRISMA guidelines (Figure 1). The authors also searched through the references listed within those published articles. The search was conducted using combinations of the terms “Novel coronavirus,” “COVID-19,” “SARS-CoV-2,” “Maternal,” “Neonatal,” “Mother,” “Pregnancy,” “Newborn,” “Infant,” “Antibody,” and “Laboratory” in the English language on July 20, 2020. Duplicate entries were searched by their titles, authors, date of publication, and Digital Object Identifier.

### Study Selection and Data Extraction

Two authors (C.Z. and J.Z.) searched the literature, compiled all articles identified through the literature search, and extracted the data. The primary eligibility for inclusion of studies were published studies, with patient pregnancy on admission and COVID-19 infection confirmed by laboratory diagnosis, which investigated maternal/neonatal complications, and recorded blood assay results. The following information was extracted from each eligible study: authors, publication date, type of study, study size, maternal characteristics (age, gestation, preterm birth, vaginal/cesarean birth, symptom severity, and maternal death), infant characteristics (birth weight, Apgar score, and neonatal death), maternal and neonatal blood assays, and laboratory-confirmed vertical transmission of COVID-19. We excluded duplicate studies, articles where full text was not available, other languages than English, opinions, and perspectives. After assessment for duplicates, titles, and abstracts, and full texts of articles, the individual patient characteristics and summary estimates from each selected article were extracted to an Excel spreadsheet (see section Data Availability Statement).

### Data Synthesis

The meta-analysis included all studies. Pooled means on age, gestational age, blood assay values, antibody levels, and pooled prevalence on preterm birth, C-section delivery, vertical transmission, abnormal blood assay values, and elevated antibody levels were assessed using the Generalized Linear Mixed model; the meta-analysis was performed using the “meta” (10) and “metaprop” (11) packages in the R statistical software (12). The random effects model was applied to calculate the pooled prevalence and single means with a 95% confidence interval (95% CI). Heterogeneity was assessed using Cochran *Q* test and Higgins' *I*^2^ statistic (represented as a percentage, using the Restricted Maximum Likelihood method). Begg's Test, Egger's test, and a new publication bias statistic, Lin and Chu (13), which generally performs better than Egger's test (14), were used for testing publication bias. Statistical significance was considered with a *P* < 0.05. For studies without reported standard deviations (SD), their SD was replaced with the average of the studies whose SD were calculated or reported (15).

## Results

### Study Characteristics

A total of 1,673 literatures were initially retrieved from searching online databases and citations. Among these, 695 duplicate literatures were identified and removed. Remaining literatures were screened according to their titles and abstracts. Twenty-nine articles were selected to be analyzed (Table 1). A total of 375 SARS-CoV-2-positive pregnant women in late pregnancy and neonates born to these infected mothers were evaluated in 4 cohort studies, 7 case series, 11 retrospectives, and 2 observational studies, which were all conducted between December 2019 and July 2020. Three studies provided antibody (IgG/IgM) test results.

**Table 1:** Baseline characteristics of included studies.

**Study**	**Study period**	**Study type**	**Study size**	**Mean age**	**Mean gestational age at birth**
Zeng et al. (16)	January to February 2020	Cohort	3	NA	37
Dong et al. (17)	February 2020	Case Study	1	29	34
Chen et al. (18)	January 2020	Retrospective	9	29.89	37.11
Zhu et al. (19)	January to February 2020	Retrospective	9	30.89	35.11
Khan et al. (20)	January to February 2020	Case Series	17	29.29	37.82
Yin et al. (21)	January to February 2020	Cohort	17	31	37
Liu et al. (22)	January to February 2020	Retrospective	18	31	38.6
Chen et al. (23)	March 2020	Case Series	4	29	37.75
Alzamora et al. (24)	March 2020	Case Study	1	41	33
Xiong et al. (25)	January 2020	Case Study	1	25	33
Yang et al. (26)	January to March 2020	Cohort	13	30.2	38.2
Hantouchzadeh et al. (27)	March 2020	Retrospective	9	34.86	30
Qiancheng et al. (28)	January to March 2020	Cohort	22	30	38
Hu et al. (29)	January to February 2020	Case Series	7	32.71	38.71
Lu et al. (30)	February 2020	Case Study	1	22	38
Yan et al. (31)	January to March 2020	Retrospective	99	30.8	38
Ferrazzi et al. (32)	March 2020	Retrospective	24	30.9	NA
Zeng et al. (33)	March 2020	Retrospective	6	NA	NA
Iqbal et al. (34)	April 2020	Case Study	1	34	NA
Yang et al. (35)	January 2020	Case Series	7	NA	36.71
Wu et al. (36)	December 2019 to March 2020	Retrospective	20	33.35	31.87
Zhang et al. (37)	January to March 2020	Observational	18	29.11	38.4
Liu et al. (38)	March 2020	Retrospective	21	31	NA
Lee et al. (39)	January 2020	Case Study	1	28	37
Li et al. (40)	January to February 2020	Retrospective	16	30.9	38
Ibrahim (41)	February 2020	Retrospective	6	NA	NA
Semeshkin et al. (42)	May 2020	Observational	20	NA	NA
Liu et al. (43)	January to February 2020	Case Series	3	32.67	39
Adhdam et al. (44)	March 2020	Case Study	1	NA	NA

### Quantitative Analysis

#### Characteristics and Blood Assay of Pregnant Women With COVID-19

**Figure 2:**
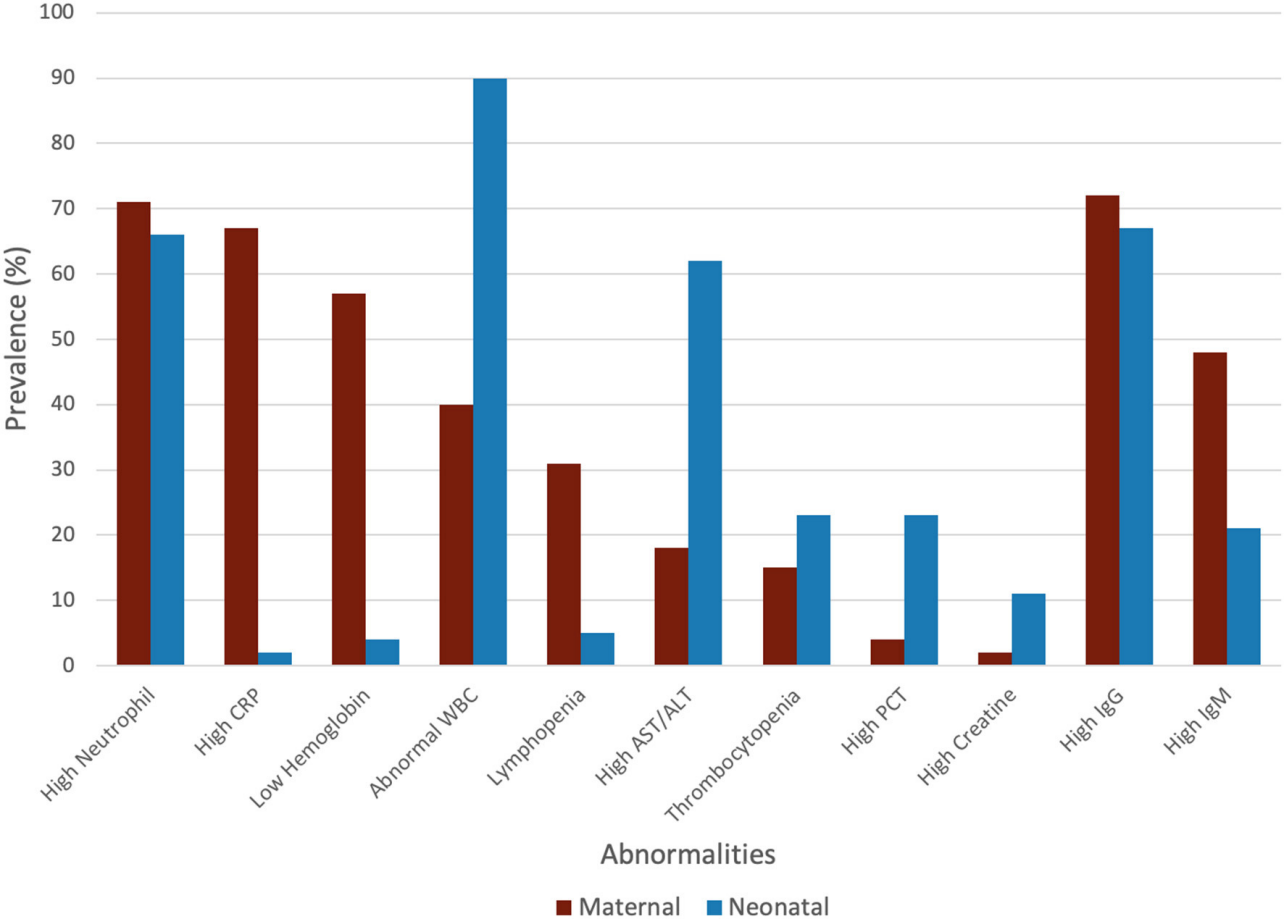
Prevalence of maternal and neonatal abnormalities.

The general characteristics of pregnant patients with COVID-19 are summarized in Table 2, and the prevalence of maternal and neonatal abnormalities are summarized in Figure 2, and their meta-regression results are summarized in Table 3. The pooled mean maternal age was estimated at 30.86 years (95% CI: 30.16–31.55). The pooled mean of gestational age was estimated as 37.20 weeks (95% CI: 36.52–37.88) and was not significantly different from the normal gestational age of 37 weeks (45). The global proportion of preterm birth was estimated at 11% before the pandemic (46). This study found the proportion to be 34.19% (95% CI: 28.29–40.61%), suggesting a trend for higher risk of preterm birth in pregnant patients with COVID-19. The results of the meta-regression did not show any correlation between the means of gestational age and maternal age (*P* = 0.1542).

**Table 2:** Meta-analysis of general maternal and neonatal characteristics.

**Statistic**	**No. of studies**	**Reference range**	**Mean/prevalence (%)**	**95% CI LB**	**95% CI UB**	***I*^2^ (%)**	***Q* test *P*-value**	**Begg's test**	**Egger's test**	**Lin and Chu**
Maternal Age (years)	23		30.86	30.16	31.55	66.3	<0.01	0.63	0.76	0.68
Gestational Age (weeks)	22	38–42	37.20	36.52	37.88	77.8	<0.01	0.09	0.02	0.03
Preterm Birth	21	10%	34.19%	28.29%	40.61%	6.3	0.06	0.30	0.95	0.68
Vaginal Birth	20	68.1%	15.33%	8.49%	26.11%	60.9	<0.01	0.21	0.31	0.02
Cesarean Birth	20	31.9%	82.69%	70.48%	90.53%	66.5	<0.01	0.36	0.41	0.07
Severe Symptoms	11		9.28%	2.83%	26.44%	75.0	<0.01	0.37	0.99	0.14
Maternal Death	26		0%	0%	100%	98.9%	0.01	0.00	0.01	0.00
Birth Weight (g)	23	2,500–4,000	3,040	2,910	3,170	74.8	<0.01	0.26	0.43	0.00
Low Birth Weight	18	8.2%	13.32%	5.92%	27.31%	57.4	<0.01	0.29	0.32	0.02

**Table 3:** Meta-analysis of correlations between maternal and neonatal characteristics.

**Moderator**	**Response variable**	**No. of studies**	**Correlation**	**95% CI LB**	**95% CI UB**	**Correlation *P-*value**	***I* ^**2**^**	***Q* test *P-*value**	**Begg's test**	**Egger's test**	**Lin and Chu**
Mat. IgG	Neo. IgG	3	0.85	0.67	0.93	<0.01	0%	0.41	0.16	0.00	0.00
Mat. IgM	Neo. IgM	3	0.43	0.06	0.70	0.03	0%	0.97	0.22	0.03	0.02
Maternal age	Gestation	20	0.15	−0.50	0.08	0.15	95%	<0.01	0.02	0.00	0.00
	Weight	20	−33.71	−87.24	19.82	0.22	80%	<0.01	0.30	0.54	0.27
	WBC	16	−0.12	−0.55	0.31	0.58	73%	0.00	0.65	0.43	0.76
	Neutrophil	11	0.55	−0.08	1.18	0.08	98%	<0.01	0.04	0.11	0.09
	Lymphocytes	13	0.05	−0.11	0.10	0.96	69%	0.00	0.67	0.56	0.90
	Platelets	9	−2.57	−9.66	4.53	0.48	79%	<0.01	0.75	0.04	0.24
	Hemoglobin	9	−0.18	−2.17	1.81	0.86	82%	0.00	0.02	0.00	0.05
	C-reactive	17	−1.22	−4.20	1.77	0.42	99%	<0.01	0.58	0.04	0.00
	Creatine-Kin.	6	−0.98	−5.64	3.69	0.68	99%	<0.01	0.70	0.05	0.23
	PCT	8	0.01	−0.08	0.11	0.26	96%	<0.01	0.70	0.57	0.12
	AST	14	0.51	−5.18	6.21	0.86	99%	<0.01	0.54	0.77	0.00
	ALT	15	0.09	−7.83	8.02	0.98	100%	<0.01	0.27	0.74	0.00
	Bilirubin	4	−1.01	−4.03	2.00	0.51	83%	0.00	0.72	0.63	0.62
	Lact Dehydro	4	10.82	47.63	69.26	0.72	98%	<0.01	0.75	0.83	0.96
	Albumin	6	−1.02	−3.48	1.43	0.41	100%	<0.01	0.42	0.46	0.51
	BUN	7	−0.03	0.23	0.17	0.78	97%	<0.01	1.00	0.43	0.68
	D-dimer	7	−0.08	−0.38	0.23	0.63	95%	<0.01	0.36	0.66	0.59
Gestational age	Weight	21	111.26	56.04	166.4	<0.01	56%	0.00	0.18	0.09	0.14
	WBC	15	−0.26	−0.81	0.30	0.37	75%	0.00	0.52	0.51	0.60
	Neutrophil	10	−1.17	−1.84	-0.49	0.00	95%	<0.01	0.05	0.03	0.14
	Lymphocytes	12	0.06	−0.14	0.26	0.58	73%	0.00	0.54	0.11	0.81
	Platelets	8	−1.04	−12.80	10.73	0.86	83%	<0.01	0.71	0.00	0.24
	Hemoglobin	8	3.25	0.54	5.97	0.02	58%	0.04	0.03	0.09	0.10
	C-reactive	16	−1.30	−6.02	3.43	0.59	99%	<0.01	0.52	0.03	0.00
	Creatine-Kin.	6	7.56	−4.63	19.74	0.22	99%	<0.01	0.70	0.14	0.23
	PCT	8	0.02	−0.15	0.18	0.83	98%	<0.01	0.70	0.03	0.12
	AST	13	−1.56	−11.27	8.15	0.75	99%	<0.01	0.39	0.77	0.01
	ALT	14	−0.82	−14.29	12.66	0.91	100%	<0.01	0.18	0.58	0.00
	Albumin	5	0.87	−0.33	2.08	0.16	92%	<0.01	0.80	0.45	0.67
	BUN	6	0.26	0.01	0.51	0.04	67%	0.03	0.44	0.68	0.90
	D-dimer	6	−0.18	−0.61	0.25	0.41	95%	<0.01	0.72	0.38	0.66
Gestational age^**^	WBC	9	0.48	−0.35	1.31	0.26	14%	0.35	0.75	0.05	0.46
	Neutrophil	5	−0.44	−1.75	0.87	0.51	26%	0.21	0.48	0.35	0.50
	Lymphocytes	6	−0.03	−0.91	0.85	0.95	92%	<0.01	1.00	0.07	0.81
	Platelets	6	3.71	−19.28	26.70	0.75	79%	0.01	1.00	0.19	0.69
	Hemoglobin	4	3.20	−5.40	11.81	4.66	42%	0.19	0.75	0.56	0.65
	C-reactive	8	−0.33	−1.00	0.35	0.34	98%	<0.01	105.00	0.79	0.60
	Creatine-Kin.	5	−139.9	−277.3	-2.48	0.05	100%	<0.01	0.48	0.36	0.70
	PCT	6	−0.16	−1.31	0.99	0.79	100%	<0.01	0.44	0.06	0.54
	AST	9	3.75	−2.21	9.70	0.22	0%	0.68	0.75	0.80	0.91
	ALT	9	2.03	−0.83	4.89	0.16	0%	0.93	0.46	0.22	0.29
	Apgar 1 min	17	0.24	−0.09	0.57	0.15	99%	<0.01	0.45	0.43	0.10
	Apgar 5 min	17	0.17	−0.15	0.49	0.31	99%	<0.01	0.41	0.87	0.00
	Vertical	22	1.06	2.95	0.82	0.26	96%	1.00	0.72	0.30	0.31
Birth weight^**^	WBC	10	0.00	0.00	0.01	0.12	11%	0.14	0.86	0.94	0.59
	Neutrophil	5	−0.01	−0.03	0.01	0.27	0%	0.03	0.46	0.45	0.50
	Lymphocytes	6	0.00	−0.01	0.01	0.82	92%	<0.01	1.00	0.30	0.81
	Platelets	7	0.03	−0.05	0.12	0.44	81%	0.00	0.77	0.65	0.73
	Hemoglobin	5	0.02	−0.01	0.04	0.30	23%	0.21	0.23	0.14	0.20
	C-reactive	9	0.00	−0.01	0.00	0.15	97%	<0.01	0.11	0.90	0.46
	Creatine-Kin.	5	0.15	−0.95	1.26	0.78	100%	<0.01	0.48	0.04	0.70
	PCT	6	−0.01	−0.01	−0.01	<0.01	90%	<0.01	0.44	0.06	0.53
	AST	9	0.02	0.00	0.05	0.09	0%	0.83	0.75	0.84	0.91
	ALT	9	0.01	−0.01	0.03	0.21	0%	0.90	0.46	0.31	0.29
	Apgar 1 min	17	0.00	0.00	0.00	0.04	98%	<0.01	0.45	0.10	0.10
	Apgar 5 min	17	0.00	0.00	0.00	0.05	99%	<0.01	0.41	0.58	0.00
	Vertical	23	0.00	−0.01	0.00	0.25	89%	0.00	0.56	0.87	0.47

** *Indicates that the corresponding response variable is a neonatal characteristic; unless specified, the corresponding response variable is a maternal characteristic*.

Frequency of severe symptoms/admission to intensive care units (ICUs) was estimated at 9.28% (95% CI: 2.83–26.44%), which is not significantly different from the normal. There were seven cases of maternal death in this review; the prevalence of maternal death was not significantly different from zero (95% CI: 0.0–100.0%) and had significant publication bias (Begg's Test *P* < 0.01; Egger's Test *P* = 0.01; Lin and Chu *P* < 0.01).

The maternal laboratory findings are summarized in Table 4. The most common laboratory findings were high neutrophil count, which was present in 71.9% of patients (95% CI: 38.49–90.87%) followed by CRP [67.7% (95% CI: 50.59–81.0)] and low hemoglobin [57.3% (95% CI: 26.0–86.76%)]. The pooled mean of neutrophils was estimated as 7.08 10^9^/L (95% CI: 5.07–9.10), CRP was estimated as 24.88 mg/L (95% CI: 13.80–35.96), and hemoglobin was estimated as 112.41 g/L (95% CI: 13.70–121.13). The results of the meta-regression showed that neutrophil levels have a significant relationship with gestational age (coefficient: −1.18, *P* < 0.01, 95% CI: −1.84–0.49; intercept = 50.48) and that hemoglobin levels have a relationship with gestational age (coefficient: 3.25, *P* = 0.02, 95% CI: 0.54–5.97; intercept = −5.35). Less commonly encountered abnormalities were lymphopenia, which was found in 31.99% of patients (95% CI: 21.64–44.49%), thrombocytopenia [15.07% (95% CI: 3.95–43.37%)], and elevated procalcitonin (PCT). The pooled prevalence of elevated PCT was estimated at 3.76% (95% CI: 0.06–72.79%) and had significant publication bias (Lin and Chu *P* = 0.01).

**Table 4:** Meta-analysis of maternal laboratory tests and antibodies.

**Statistic**	**No. of studies**	**Reference range**	**Mean/prevalence (%)**	**95% CI LB**	**95% CI UB**	***I*^**2**^ (%)**	***Q* test *P*-value**	**Begg's test**	**Egger's test**	**Lin and Chu**
WBC (10^9^/L)	15	4.5–11	8.50	7.60	9.43	61.2	<0.01	0.63	0.76	0.68
Normal WBC	16		60.32%	47.28%	72.04%	59.2	<0.01	0.09	0.02	0.03
Neutrophil (10^9^/L)	11	1.5–8.0	7.08	5.07	9.10	99.7	0.00	0.30	0.95	0.68
High neutrophil	6		71.39%	38.49%	90.87%	60.9	0.02	0.21	0.31	0.02
Neutrophil percent	5	45–75%	78.47	71.69	85.24	90.0	<0.01	0.36	0.41	0.07
Lymphocytes (10^9^/L)	13	1.0–5.0	1.26	1.14	1.39	62.7	<0.01	0.37	0.99	0.14
Low lymphocytes	15		31.99%	21.64%	44.49%	59.4	<0.01	0.00	0.01	0.00
Lymphocytes percent	6	18%−45%	25.55	4.67	46.43	100	0.00	0.65	0.51	0.18
Platelets (10^3^/μl)	9	150–450	177.92	151.02	204.82	77.3	<0.01	0.85	0.05	0.09
Low platelets	8		15.07%	3.95%	43.37%	68.1	<0.01	0.04	0.01	0.15
Hemoglobin (g/L)	9	120–155	112.4	13.70	121.1	70.2	<0.01	0.44	0.21	0.68
Low hemoglobin	7		57.30%	26.00%	86.76%	57.3	<0.01	0.41	0.99	0.91
CRP (mg/L)	17	<10	24.88	13.80	35.96	97.1	<0.01	0.67	0.49	0.06
Elevated CRP	13		67.70%	50.59%	81.09%	70.0	<0.01	0.58	0.26	0.93
Creatine-kinase (U/L)	6	22–198	49.56	26.34	72.79	99.2	<0.01	1.00	0.26	0.46
High creatine-kinase	5		2.17%	0.31%	13.88%	0.0	0.8267	0.75	0.12	0.40
PCT (ng/ml)	8	<0.5	0.27	0.00	0.59	80.3	<0.01	0.88	0.59	0.53
High PCT	8		3.76%	0.06%	72.79%	70.5	0.04	0.02	0.02	0.94
AST (U/L)	14	<36	41.11	18.24	63.98	96.6	<0.01	0.19	0.10	0.09
ALT (U/L)	15	<36	40.39	9.00	71.78	98.9	<0.01	0.58	0.01	0.01
Elevated AST/ALT	13		18.81%	8.21%	37.49%	68.0	<0.01	0.29	0.04	0.00
Total bilirubin (μmol/L)	4	1–12	14.37	2.98	25.76	77.5	<0.01	0.70	0.09	0.61
Lactate dehydro. (U/L)	4	<225	236.9	148.3	325.5	97.4	<0.01	0.07	0.20	0.02
Albumin (g/L)	6	35–50	26.73	16.85	36.07	99.3	<0.01	0.70	0.19	0.12
BUN (mmol/L)	7	2.5–7.1	3.22	2.28	4.14	88.5	<0.01	1.00	0.31	0.01
PT (s)	3	8.7–11.5	12.11	11.72	12.50	0.0	<0.01	0.54	0.08	<0.01
APTT (s)	3	30–40	3.10	29.93	44.27	90.9	<0.01	0.27	0.10	0.00
D-dimer (mg/L)	7	<0.5	2.27	1.30	3.23	95.5	<0.01	0.62	0.95	0.15
IgG (AU/ml)	15	<10	76.90	43.02	110.79	61.2	<0.01	0.72	0.86	0.50
High IgG	16		72.73%	55.35%	85.16%	59.2	<0.01	0.50	0.52	0.17
IgM (AU/ml)	11	<10	96.89	0.00	212.81	99.7	0.00	0.42	0.34	0.48
High IgM	6		48.94%	14.35%	84.58%	60.9	0.02	1.00	0.85	0.66

The following outcomes were from a meta-analysis with <10 studies. The pooled mean of lactate dehydrogenase has been estimated as 236.91 U/L (95% CI: 148.28–325.54), which is not significantly different from the normal. The results of the meta-regression showed that blood urea nitrogen (BUN) levels have a significant relationship with gestational age (coefficient: 0.26, *P* = 0.04, 95% CI: 0.01–0.51). The pooled mean of prothrombin time (PT) was estimated as 12.11 s (95% CI: 11.7175–12.4990), which is significantly higher from the normal (8.7–11.5). The pooled mean of D-dimer has been estimated as 2.2651 mg/L (95% CI: 1.2956–3.2346), which is significantly higher than the normal range (<0.5).

#### Characteristics and Blood Assay of Infants Born to Pregnant Women With COVID-19

The general characteristics of infants born to mothers with COVID-19 are summarized in Table 2, and their meta-regression results are summarized in Table 3. The pooled mean of birth weight has been estimated as 3,040 g (95% CI: 2,910–3,170). The pooled prevalence of low birth weight has been estimated as 13.2% (95% CI: 5.92–27.31%), which is not significantly different from the estimated worldwide prevalence of low birth weight in 2015 of 14.5% (47). The results of the meta-regression showed that the means of birth weight have a relationship with gestational age with a correlation of 111 g/week (*P* < 0.01, 95% CI: 56.04–166.48; intercept = −1,106.99). This is not significantly from previous studies (48).

The neonatal laboratory findings are summarized in Table 5. The most common laboratory findings were elevated IL-6, which was present in 91.1% of the neonates (95% CI: 32.38–99.97%), abnormal white blood cell count (WBC), elevated neutrophil count [66.67% (95% CI: 47.33–81.66%)], and elevated AST/ALT [62.0% (95% CI: 47.96–74.28%)]. The pooled prevalence of normal WBC has been estimated as 9.85% (95% CI: 3.13–26.96%), indicating that over 90% of all patients had abnormal white blood cell counts. This can be attributed to the high neutrophil counts in neonates, which was estimated at 10.18 × 10^9^/L (95% CI: 8.90–11.46). The pooled mean of IL-6 levels was estimated at 23.22 pg/ml (95% CI: 16.94–29.49), which is significantly higher than the normal. AST levels were estimated at 59.34 U/L (95% CI: 49.81–68.86), which is also significantly higher than the normal.

**Table 5:** Meta-analysis of neonatal laboratory tests and antibodies.

**Statistic**	**No. of studies**	**Reference range**	**Mean/prevalence (%)**	**95% CI LB**	**95% CI UB**	***I*^**2**^ (%)**	***Q* test *P-*value**	**Begg's test**	**Egger's test**	**Lin and Chu**
WBC (10^9^/L)	10	4.5–11	3,040	2,910	3,170	41.9%	0.08	0.26	0.43	0.00
Normal WBC	10		13.32%	5.92%	27.31%	22.6%	0.17	0.29	0.32	0.02
Neutrophil (10^9^/L)	5	1.5–8.0	14.73	13.05	16.42	17.2%	0.31	0.86	0.94	0.60
High neutrophil	5		9.85%	3.13%	26.96%	0.0%	0.21	0.12	0.69	0.02
Neutrophil (%)	3	45%−75%	10.18	8.90	11.46	96.7%	<0.01	0.33	0.32	1.00
Lymphocytes (10^9^/L)	6	1.0–5.0	66.67%	47.33%	81.66%	94.5%	<0.01	0.45	0.85	0.93
Low lymphocytes	8		60.09	40.45	79.73	75.9%	<0.01	1.00	0.86	0.98
Lymphocytes (%)	4	18%−45%	2.87	1.57	4.16	89.7%	<0.01	0.85	0.15	0.00
Platelets (10^3^/μl)	7	150–450	4.75%	0.35%	41.35%	72.5%	<0.01	1.00	0.03	0.01
Low platelets	7		28.85	15.26	42.45	21.2%	0.22	0.28	0.49	0.96
Hemoglobin (g/L)	5	120–155	250.8	219.4	282.2	34.7%	0.19	0.65	0.84	0.81
Low hemoglobin	5		23.37%	11.27%	42.26%	0.0%	0.73	0.36	0.31	0.66
CRP (mg/L)	9	<10	168.9	158.1	179.7	96.8%	<0.01	0.14	0.14	0.73
Elevated CRP	9		4.17%	0.58%	24.35%	0.0%	0.98	0.14	0.66	0.01
Creatine-kinase (U/L)	5	22–198	1.86	0.56	3.15	99.6%	<0.01	0.11	0.24	0.37
High creatine-kinase	5		1.96%	0.28%	12.65%	69.4%	0.02	0.00	0.04	0.00
PCT (ng/ml)	6	<0.5	64.84	61.03	68.65	50.6%	0.09	0.33	0.27	0.56
High PCT	6		10.65%	1.30%	51.92%	23.0%	0.21	0.62	0.92	0.88
AST (U/L)	9	<36	0.29	0.22	0.35	0.0%	0.60	0.44	0.18	0.14
ALT (U/L)	9	<36	23.17%	8.40%	49.78%	0.0%	0.82	1.00	0.20	0.02
Elevated AST/ALT	8		59.34	49.81	68.86	0.0%	0.80	0.75	0.79	0.05
Albumin (g/L)	3	35–50	14.65	11.93	17.37	44.7%	0.16	0.46	0.16	0.00
IL-6 (pg/ml)	3		62.00%	47.96%	74.28%	35.6%	0.21	0.71	0.72	0.23
High IL-6	3		32.94	28.67	37.21	86.1%	<0.01	0.60	0.71	0.81
IgG (AU/ml)	4	<10	23.22	16.94	29.49	89.2%	<0.01	0.60	0.47	0.33
High IgG	4		91.10%	32.38%	99.97%	2.0%	0.30	0.60	0.27	0.17
IgM (AU/ml)	4	<10	72.47	26.41	118.5	91.6%	<0.01	0.04	0.00	0.99
High IgM	4		67.16%	37.87%	87.27%	72.5%	<0.01	0.70	0.12	0.00
Apgar, 1 min	20	7–10	15.86	0.00	34.65	96.0%	<0.01	0.17	0.16	0.84
Apgar, 5 min	20	7–10	20.65%	2.75%	70.54%	97.6%	<0.01	0.70	0.85	0.83
Vertical transmission	27		7.94	7.26	8.62	88.6%	<0.01	0.87	0.75	0.01

Frequency of high PCT was less common and has been estimated as 23.17% (95% CI: 8.4–49.785%). The results of the meta-regression showed that PCT levels have a relationship with birth weight (coefficient: −0.01, *P* < 0.01, 95% CI: −0.01 to 0.01; intercept = 16.66).

The pooled mean of 1-min Apgar has been estimated as 7.94 (95% CI: 7.26–8.62). The results of the meta-regression showed that 1-min Apgar scores have a relationship with birth weight (coefficient: 0.002, *P* = 0.04, 95% CI: 0.00–0.01; intercept = 1.26). The pooled mean of 5 min Apgar has been estimated as 8.97 (95% CI: 8.34–9.61). The results of the meta-regression showed that 5 min Apgar scores have a relationship with birth weight (coefficient: 0.002, *P* = 0.04, 95% CI: 0.00–0.004; intercept = 2.49).

The pooled prevalence of vertical transmission has been estimated as 0.18%, but it was not significantly different from zero (95% CI: 0.0%−8.95%; *I*^2^ = 88.6%, *P* < 0.01), and had significant publication bias (Begg's Test *P* < 0.01; Lin and Chu *P* < 0.01).

#### Antibodies of Infants and Pregnant Women With COVID-19

High IgG in mothers was highly prevalent and has been estimated as 72.73% (95% CI: 55.35–85.16%). The pooled mean of maternal IgG was estimated as 76.90 AU/ml (95% CI: 43.02–110.79), which is significantly higher than the normal. The pooled prevalence of high IgM in mothers was less than high IgG and was estimated as 48.94% (95% CI: 14.35–84.58). The pooled mean of maternal IgM was estimated as 96.89 AU/ml (95% CI: 0.00–212.81).

High IgG in neonates born to mother with COVID-19 was highly prevalent and was estimated as 67.16% (95% CI: 37.87–87.27%). The pooled mean of neonatal IgG was estimated as 72.47 AU/ml (95% CI: 26.41–118.54). The pooled prevalence of high IgM in neonates was estimated as 20.65% (95% CI: 2.75–70.54%). The pooled mean of neonatal IgM was estimated as 15.86 AU/ml (95% CI: 0.00–34.65).

The results of the meta-regression showed that infant IgG levels have a relationship with their mother's IgG level (coefficient: 0.85, *P* < 0.01, 95% CI: 0.67–0.93) and that infant IgM levels have a relationship with their mother's IgM level (coefficient: 0.43, *P* = 0.03, 95% CI: 0.06–0.70).

## Discussion

### Characteristics and Blood Assay of Pregnant Women With COVID-19

The global proportion of cesarean section was ~21.1% in 2017 (49). The present study found the overall proportion of cesarean section to be 82.7%, much higher than the global prevalence. While the decision to undergo C-section can vary due to differences in clinical practice and accepted standards of care, reasons found in studies selected in this review suggest that COVID-19 patients are more likely to have a C-section because they had underlying disease and intolerance against respiratory dysfunction. Given this is a novel disease, there is a general tendency for clinicians to avoid more complicated deliveries by undergoing C-section.

Results from our study suggest that severe symptoms or the need for intensive care appeared to be higher than that that for non-pregnant women of similar age (30.56 years). We found the proportion to be 9.3%, which was higher than previous estimates of 4.2–7.0% (50, 51); however, severe symptom criteria and ICU admission criteria were not well defined and varied between studies. Furthermore, this study's prevalence of severe symptoms is not significantly different from previous estimates, or from the general prevalence of severe COVID-19. Therefore, there is not enough evidence to suggest pregnant women are at increased risk of ICU admission or more severe symptoms. Prevalence of maternal death was not significantly different from zero; all maternal deaths were from a single study (27), so maternal death is much more likely due to the quality of birthing conditions than due to maternal complications caused by COVID-19.

The most common abnormalities found in the pooled blood assay were high neutrophil count, increased C-reactive protein (CRP), and low hemoglobin (71.9, 67.7, and 57.3%, respectively). Compared to abnormalities found in previous reviews, the only abnormalities shared by our review was increased CRPs among mothers with COVID-19 (2, 8, 9, 50). Furthermore, the pooled mean of CRP was significantly higher than the normal range (<10 mg/L) for healthy non-pregnant patients, indicating that elevated CRP is strongly correlated with infection. Our mean CRP level and elevated CRP prevalence is not significantly different from recent meta-analyses on non-pregnant COVID-19-positive patients (52–54). This is expected since COVID-19 can cause an overactive immune response, and CRP is a marker of that increased inflammation throughout the body.

Thrombocytopenia was previously identified as a symptom of infection. In this current study, the prevalence of low platelet counts was estimated at 15.1%, and its pooled mean was not significantly different from the normal range (150–450 10^3^/L). Platelet counts generally decrease during pregnancy, particularly during the third trimester, termed “gestational thrombocytopenia.” In order to adjust for this, we used a lower limit for platelet count of 115 10^3^/μl (55). Using this new parameter, a loose interpretation could be that COVID-19 does not significantly worsen gestational thrombocytopenia since the mean platelet levels is not significantly lower than the normal for pregnant patients. Our platelet results also did not differ significantly from those of a recent meta-analysis on non-pregnant COVID-19-positive patients, so pregnant patients are at no greater risk.

Similarly, while elevated lactate dehydrogenase was identified in COVID-19 patients in previous studies and is associated with worse clinical outcome (53), our results showed that its pooled mean was not significantly different from the normal range (<225 U/L). However, due to the low number of studies that included lactate dehydrogenase in their blood assay, it is not possible to refute if it could also be correlated with infection.

Interestingly, we did not find elevated PCT to be a prevalent symptom of infection. Its prevalence was estimated at 3.8%, and its pooled mean, 0.29 ng/ml, was not significantly different from the normal range (<0.15 ng/ml). Furthermore, there was significant publication bias (Lin and Chu *P* < 0.01) for the pooled prevalence, so it is likely that the true prevalence is lower than the one presented in this study due to the inflation of elevated PCT publications (56). This provides more evidence that suggests that elevated PCT is not a prevalent symptom of COVID-19.

In this study, we present other noteworthy values from the blood assays: neutrophils count, D-dimer, hemoglobin, and BUN. The most prevalent abnormality was high neutrophil count, or neutrophilia, with 71.4% of the women having this condition. Neutrophilia is its associated increased risk of small-for-gestational-age (SGA) birth, which reflects a cycle of inflammation and placental insufficiency (57). The mean neutrophil level was estimated at 7.08 × 10^9^/L, which is over twice as concentrated than in non-pregnant COVID-19-positive patients (53), and the prevalence of neutrophilia is over 10 times greater (52). The results of the meta-regression further show that there is a significant negative correlation (*P* < 0.01) between a mother's neutrophil count and the gestational age at birth, with a gestational age of 32 weeks corresponding to neutrophil count of 13.1 × 10^9^/L. Therefore, excessively high neutrophil counts during mid-to-late pregnancy could be a risk indicator for preterm delivery. This is further supported by the high mean neutrophil–lymphocyte ratio (NLR), which was found to be 5.60. High NLR has been associated with greater risk of preeclampsia (58) and preterm birth (59).

The pooled mean of D-dimer was estimated as 2.27 mg/L, which is much greater than the normal range (<0.5 mg/L) for non-pregnant patients. D-dimer is generally elevated during pregnancy; therefore, using this upper limit of 0.5 mg/L would result in false positives if using an upper limit of 0.5 mg/L. Studies have suggested increasing this threshold to 1.0 or 2.0 mg/L (60, 61). Still, the point estimate and confidence interval (1.30–3.23) are still above these elevated thresholds. Our pooled mean for D-dimer was over twice as concentrated than in non-pregnant COVID-19-positive patients (53). D-dimer is a biomarker for disease severity and blood clotting, so these observations provide evidence that indicate risk of venous thromboembolism and/or pulmonary embolism in mothers with COVID-19.

Low hemoglobin levels were the second most prevalent abnormality in the present study, with 57.3% patients presenting this abnormality. In general, pregnancy-induced anemia is common, so the normal hemoglobin range for pregnant women in the third trimester is 95–150 g/L (62). However, even given this lower threshold, there were many studies that included pregnant women with very low hemoglobin levels. This is concerning since very low hemoglobin levels are associated with increased fetal risk (63). Furthermore, the results from the meta-regression found a significant positive relationship (*P* = 0.02) between hemoglobin levels and gestational age, with the gestational age of 32 weeks corresponding to 98.7 g/L. Very low hemoglobin levels during mid-to-late pregnancy could be a risk indicator for preterm birth, and the infant should be prioritized in consideration for neonatal ICU.

The pooled mean of BUN was estimated as 3.22 mmol/L, which falls within the normal range (2.5–7.1). We found that BUN had a significant positive correlation (*P* = 0.04) with gestational age, with the gestational age of 32 weeks corresponding to 1.44 mmol/L. However, a previous study (whose sampling frame was all pregnant women, not just those infected with COVID-19) found a negative correlation between BUN and gestational age, with a gestational age of 32 weeks corresponding to 17.1 mmol/L (64). Since preliminary studies found that elevated BUN levels increase risk of in-hospital death by 2.51 in COVID-19 patients (65), it is possible that both of these conclusions are true: when BUN levels are abnormally low (<1.5 mmol/L) and are increasing, risk of kidney damage decreases, but the risk increases after BUN levels exceed 4 mmol/L (which is the upper bound for our confidence interval).

### Characteristics and Blood Assay of Infants Born to Pregnant Women With COVID-19

The most common abnormality found in the pooled blood assay was elevated IL-6, abnormal white blood cell count (WBC), elevated neutrophil count, and elevated AST/ALT. The pooled mean of IL-6, 23.22 pg/ml, is significantly above the normal range for IL-6 (5–15 pg/ml) (66). Furthermore, the pooled prevalence of elevated IL-6 is 91.1%, which is very frequent. Since elevated IL-6 has been deemed a valid marker for predicting neonatal sepsis (NS) (67), more blood assays for neonates should include IL-6 as a tool for early NS diagnosis. Abnormally elevated IL-6 levels without IL-10 regulation (elevated IL-10 levels) are an essential indicator for further neonatal complications such as necrotizing enterocolitis (NEC) (68). However, only a single study selected for this meta-analysis reported IL-10, and the neonate had elevated IL-10 levels and no NEC, so this study cannot meaningfully comment on the regulation of IL-10. However, given that IL-6 is the main cytokine responsible in the COVID-19-induced cytokine storms, these cytokines could have been shared from the mother to the fetus via the placenta.

Abnormal WBC and elevated neutrophils are well recognized within neonates (69). Hyperleukocytosis (WBC > 100 × 10^9^/L) would be cause for concern, but no cases of hyperleukocytosis were identified in the present study. Similarly, the high prevalence of elevated AST/ALT in neonates has been deemed a benign condition that usually resolves within a year (70).

The pooled mean for PCT has been estimated at 0.23 ng/ml and is significantly higher than the normal value for children older than 72 h (0.15 ng/ml). The results of the meta-regression found a significant negative relationship (*P* < 0.01) between birth weight and PCT levels, corresponding to a birth weight of 2,500 g to 4.4 ng/ml. This is highly concerning because elevated PCT is a biomarker that is much more specific than any other proinflammatory marker in identifying sepsis. Using this relationship, infants with birth weights under 3,000 g could have PCT levels >2 ng/ml, indicating severe sepsis and high risk of developing organ dysfunction.

The pooled 1 and 5 min Apgar scores, 7.94 and 8.97, respectively, were not significantly different from the normal range (7–10). As expected, the 1 and 5 min Apgar scores had significant positive relationships (*P* = 0.04, *P* = 0.04, respectively) with birth weight.

The pooled prevalence of vertical transmission was estimated to be 0.18%, which is not significantly different from 0%. Therefore, we found that the risk of vertical transmission is very low. Due to the significant publication bias, this suggests that the prevalence of vertical transmission is even lower than 0.18%. Recent studies into vertical transmission of SARS-CoV-2 via the placenta have also concluded that the virus very rarely infects the placenta and can only do so with very high maternal viral loads (6). Even after placental infection, the virus may still be blocked from vertically transmitting (7). Furthermore, we found that vertical transmission does not have significant correlations with gestational age and birth weight, so positive vertical transmission is much more likely due to birthing conditions (such as cleanliness and ventilation) than due to maternal or neonatal characteristics.

### Antibodies of Infants and Pregnant Women With COVID-19

High levels of IgM antibodies were indicated as the first line of defense to SARS-CoV-2 when the disease is still active, whereas detection of SARS-CoV-2 virus IgG indicates recovery or past exposure to the virus (71). In this study, we used 10 AU/ml as the threshold for IgG/IgM detection (33). As expected, elevated IgG was highly prevalent in pregnant women with COVID-19, estimated as 72.7%; the pooled mean for IgG was significantly different from the normal range, estimated as 76.90 AU/ml.

Interestingly, 67.1% of infants born to mothers with COVID-19 had elevated IgG levels. The pooled mean for neonatal IgG was significantly different from the normal range, estimated at 72.47 AU/ml. This indicates that infants with mothers with COVID-19 may gain natural passive immunity through IgG crossing the placenta during late pregnancy (72). IgM has a larger molecular structure, making it more difficult to cross the placenta (72), though not impossible; this is reflected in the lower prevalence of elevated IgM levels (20.6%). Furthermore, the transfer of antibodies across the placenta is supported by the results of the meta-regression since there are significant positive relationships between maternal IgG and their neonate's IgG, and between maternal IgM and their neonate's IgM (*P* < 0.01, *P* = 0.03 respectively).

### Limitations

The major limitations of this systematic review are the retrospective design in almost all of the included studies, the lack of universal testing for COVID-19, the lack of standardized management and timing of women with COVID-19 and the inconsistent treatment and reporting for their newborns, and the lack of standardized blood testing. A significant proportion of the pregnancies were affected by COVID-19 during the third trimester, so we cannot meaningfully comment on early exposure. While common outcomes in blood assay, such as WBC and CRP, are commonly reported, other factors such as lactate dehydrogenase, BUN, D-dimer, and all neonatal outcomes should be tested more often so we can better verify if they are good tools to predicting the symptoms of COVID-19. Lastly, our review did not include studies that were recently published in the literature, particularly in languages other than English.

### Conclusions

This systematic review and meta-analysis corroborated with previous studies that pregnant women with COVID-19 are at higher risk of preterm birth, are more likely to undergo cesarean section, and have elevated CRP levels and prolonged PT. In contrast to previously published reviews, we did not find an association between COVID-19 and thrombocytopenia, elevated lactate dehydrogenase, and elevated PCT to be prevalent symptoms for COVID-19. We report additional findings associated with COVID-19-infected mothers, including high neutrophil counts, low hemoglobin, and risk of preterm birth. Consistent with other reports, we found little evidence for vertical transmission. In neonates, we observed that infants born to mothers with COVID-19 are more likely to have elevated PCT levels and NS, but also may gain passive immunity to COVID-19 through antibody transfer via placenta. More testing and laboratory data are needed to clarify the relationships we found between D-dimer and thromboembolism, and between BUN and gestational age. Since the evidence is still increasing, this review provides information that can guide future systematic reviews for more meaningful results and can guide current health care during the current SARS-CoV-2 pandemic.

## Data Availability Statement

The datasets presented in this study can be found in online repositories. The names of the repository/repositories and accession number(s) can be found below: Mendeley Data: http://dx.doi.org/10.17632/fh557t857g.1.

## Author Contributions

CZ and HC conceived the study. CZ and JZ searched the literature and extracted the data. CZ performed the statistical analysis and drafted the manuscript. YV and HC helped to edit the manuscript. All authors contributed to the article and approved the submitted version.

## Conflict of Interest

The authors declare that the research was conducted in the absence of any commercial or financial relationships that could be construed as a potential conflict of interest.
